# Epigenetic biomarkers and the progression of Barrett’s oesophagus to oesophageal adenocarcinoma: a systematic review protocol

**DOI:** 10.1186/s13643-026-03256-0

**Published:** 2026-07-08

**Authors:** David N. Johnston, Helen G. Coleman, Amy Jayne McKnight, Richard C. Turkington

**Affiliations:** 1 Centre for Public Health, Queen’s University Belfast, Belfast, UK; 2 The Patrick G Johnston Centre for Cancer Research, Queen’s University Belfast, Belfast, UK

**Keywords:** Barrett’s oesophagus, Biomarkers, Epigenetics, Methylation, Oesophageal adenocarcinoma, Systematic review

## Abstract

**Background:**

Oesophageal adenocarcinoma (OAC) is often diagnosed at a late stage and has a poor survival rate. Barrett’s oesophagus (BO) is a precursor to OAC, and progression occurs in a stepwise fashion, although the annual risk of progression is relatively low. The incidence of both BO and OAC have been increasing in the Western world. At present, guidelines advise that patients with BO should undergo regular endoscopic surveillance with biopsies, with the degree of dysplasia on biopsy being used to guide further management. However, there are potential disadvantages of this surveillance approach. For example, it is invasive and expensive, and there is interobserver variability between pathologists in the assessment of dysplasia. There is an urgent clinical need for new approaches to identify which patients with BO are at high risk of progression. Biomarkers could potentially be used to risk-stratify patients with BO. Epigenetic changes can occur early in the neoplastic progression of BO, making them attractive for use as potential biomarkers.

**Methods:**

A systematic review will be performed to investigate whether epigenetic biomarkers, including DNA methylation, histone modification, and non-coding RNAs, can predict the risk of progression of patients with BO to high-grade dysplasia or OAC. The electronic databases Ovid MEDLINE, Embase, and Web of Science Core Collection will be searched for terms relating to biomarkers, methylation, epigenetics, BO, and OAC. Observational (case-control, retrospective cohort, and prospective cohort) studies and interventional studies will be eligible for inclusion. Studies will be screened independently by two reviewers, and relevant data will be extracted from eligible studies. The risk of bias will be assessed for each study. Random effects meta-analyses will be carried out if possible, otherwise a synthesis without meta-analysis will be performed.

**Discussion:**

This systematic review aims to assess whether epigenetic biomarkers can predict progression of BO to OAC. This is an important clinical question, with national gastroenterology societies advising that risk stratification biomarkers could significantly improve the care of patients with BO.

**Systematic review registration:**

This systematic review protocol has been registered in PROSPERO (international prospective register of systematic reviews), with registration number CRD42024509643.

**Supplementary Information:**

The online version contains supplementary material available at https://doi.org/10.1186/s13643-026-03256-0.

## Background

Barrett’s oesophagus (BO) is defined as replacement of any portion of the distal oesophageal squamous epithelial lining by metaplastic columnar epithelium which is endoscopically visible at least 1 cm above the gastro-oesophageal junction and confirmed histopathologically from oesophageal biopsies [1]. BO is caused by chronic acid exposure of the distal oesophagus, which induces inflammation and columnar metaplasia [2], and it is the precursor to the development of oesophageal adenocarcinoma (OAC) [3, 4]. The incidence of BO has been rising in Western nations [5–7], with a study from our group showing a 159% increase in incidence between 1993–1997 and 2002–2005, not accounted for by a rise in endoscopies [7]. This is correlated with an increasing incidence of OAC [8]. Worldwide, between 1990 and 2012, the average annual percentage increase in age-standardised incidence rates for OAC was 5.2% [9], and the incidence rates of OAC are expected to continue to rise across high-income countries [10]. Globally, in 2020, OAC had an age-standardised incidence rate of 0.9 per 100,000 person-years [11]. OAC has a poor prognosis, with a 3-year age-standardised net survival of 22.8% for patients diagnosed between 2010 and 2014 in the UK [12]. Stage at diagnosis is the most important prognostic factor [11]. Most cases are diagnosed at an advanced stage, when curative treatment is not possible [11]. In the UK between 2012 and 2014, at the point of diagnosis, 13.6% of patients with OAC had localised disease, 44.0% had regional disease, and 42.4% had distant disease [13]. Patients with localised disease had a 3-year net survival of 66.0%, compared to 3.8% for patients with distant disease [13].

BO can be categorised on the degree of dysplasia, according to the Vienna classification of gastrointestinal epithelial neoplasia [14, 15]: 1. negative for dysplasia, 2. indefinite for dysplasia, 3. low-grade dysplasia, 4a. high-grade dysplasia, 4b. intramucosal carcinoma, and 5. submusocal invasion by adenocarcinoma [1]. Progression is stepwise, with the risk of progression to adenocarcinoma rising with the degree of dysplasia [16] as the epithelium accumulates molecular abnormalities [17]. A population-based study in Northern Ireland examining patient data between 1993 and 2008 found that the risk of BO progressing to either high-grade dysplasia or OAC was 0.22% per year [18].

Guidelines recommend surveillance for BO, consisting of endoscopic monitoring with biopsies taken for histopathological assessment of dysplasia [1, 19, 20]. This aims to detect dysplasia or adenocarcinoma at a curable stage [1]. Guidelines take into account the presence of intestinal metaplasia, degree of dysplasia, and length of segment in determining surveillance intervals [1]. There are several potential limitations of the current surveillance method. Firstly, it is invasive and expensive [21]. Secondly, dysplasia can be patchy and is often not obvious endoscopically; therefore, biopsies taken may miss dysplastic areas [22]. Thirdly, there is interobserver variability between pathologists when determining the degree of dysplasia [23–25], which involves subjective criteria [26]. Finally, endoscopists may also not be fully adhering to the surveillance guidelines. A study from the USA found the recommended biopsy protocol was adhered to in only 51.2% of cases, with non-adherence being associated with reduced dysplasia detection [27].

Current guidelines have suggested that a priority should be better risk stratification biomarkers to augment or replace histopathological assessment of dysplasia [1]. Biomarkers could provide an objective measurement of BO molecular changes, which correlate with degree of dysplasia, and indeed sometimes precede it [21]. Many potential biomarkers have been studied to predict the progression of BO [21], including genomic instability, DNA content abnormalities, clonal diversity, and epigenetic changes [28]. National guidelines recommend considering immunohistochemistry for p53 as an adjunct to routine clinical diagnosis as it may improve the diagnostic reproducibility of a dysplasia diagnosis [1]. However, no other biomarkers or biomarker panels are ready for clinical practice [14], due to a lack of randomised controlled evidence [1].

Epigenetics refers to heritable alterations in gene expression that are not caused by changes in the DNA sequence [29]. Epigenetic mechanisms control the transcriptional availability of genetic information [30], thus influencing gene expression in both normal cells and cancer [29]. Epigenetic modifications can induce hallmark cancer cell behaviours, promoting carcinogenesis [30]. These can occur early in neoplastic transformation, making them attractive as potential biomarkers [31]. Types of epigenetic modifications include DNA methylation, histone modification, and non-coding RNAs [29, 32]. Epigenetic factors are stable in fluids and tissue samples [33, 34], which is advantageous for their potential use as biomarkers. Given the need for better risk stratification biomarkers in BO, and the potential utility of epigenetic biomarkers, our systematic review will focus on epigenetic biomarkers and the progression of BO.

### Objectives

The objective of this systematic review is to investigate whether epigenetic biomarkers can predict the risk of progression of patients with BO to high-grade dysplasia or OAC.

## Methods

This systematic review protocol has been registered in PROSPERO (international prospective register of systematic reviews), with registration number CRD42024509643. The protocol has been developed in accordance with the Preferred Reporting Items for Systematic Review and Meta-Analysis Protocols (PRISMA-P) statement [35]. A populated PRISMA-P checklist has been provided as Additional file 1. The completed systematic review will be reported according to the Preferred Reporting Items for Systematic Reviews and Meta-Analyses (PRISMA) 2020 statement [36]. Any important amendments to the protocol will be documented on the PROSPERO record and in the completed systematic review.

### Eligibility criteria

#### Participants/population

We will include studies examining patients with BO that is non-dysplastic, has low-grade dysplasia, or is indefinite for dysplasia at baseline.

#### Interventions/exposures

We will include studies which assess any type of epigenetic biomarker, including DNA methylation, any type of histone modification, and any type of non-coding RNA. Epigenetic biomarkers will be eligible for inclusion regardless of their source, for example, both tissue-based and blood-based epigenetic biomarkers will be eligible for inclusion. Non-epigenetic biomarkers, such as genetic biomarkers, will be excluded.

#### Comparator(s)/control(s)

Studies eligible for inclusion will compare measurements of epigenetic biomarkers in patients with BO who progressed to high-grade dysplasia or OAC with measurements of epigenetic biomarkers in patients with BO who did not progress.

#### Outcome(s)

Studies assessing the progression of patients with BO which is non-dysplastic, has low-grade dysplasia, or is indefinite for dysplasia to high-grade dysplasia or OAC will be eligible for inclusion.

#### Study design

Observational (case-control, retrospective cohort, and prospective cohort) studies and interventional studies will be eligible for inclusion.

#### Setting

Studies in any setting will be eligible for inclusion provided that they satisfy the inclusion criteria.

#### Time frame

Studies which assess the progression of BO which is non-dysplastic, has low-grade dysplasia, or is indefinite for dysplasia to high-grade dysplasia or OAC will be eligible for inclusion, regardless of the duration of follow-up. Included studies should ideally follow up patients for a minimum of 12 months following their initial diagnosis with BO, as high-grade dysplasia or OAC that is detected within 12 months may have been present but not detected at the time of the initial diagnosis [37, 38].

#### Report eligibility criteria

Original research papers, abstracts and letters will be eligible for inclusion. Editorials, commentaries, review articles, systematic reviews, meta-analyses, case reports, and studies of any type focussing on non-human subjects will be excluded. Studies in all languages will be eligible for inclusion. There will be no restrictions based on year of publication or geographical location of study.

### Information sources

The electronic databases Ovid MEDLINE, Embase, and Web of Science Core Collection will be searched for subject headings and keywords relating to biomarkers, methylation, epigenetics, Barrett’s oesophagus, and oesophageal adenocarcinoma, to identify relevant publications. All databases will be searched from their inception date until the date of search. Review articles and non-human studies will be excluded as part of the search strategy where possible. No other restrictions will be applied. Reference lists of identified publications will be searched to identify possible additional relevant publications. The search will be carried out by author DNJ. The search strategy and search terms will be reviewed by a medical librarian with expertise in conducting systematic reviews. Following completion of these searches, a supplementary search of ClinicalTrials.gov will also be undertaken to identify any additional relevant records.

### Search strategy

The search strategy will have three main concepts: BO, epigenetic biomarkers, and OAC. The Boolean operator “or” will be used to search for related terms within each concept. Truncation and wildcards may be used to assist with this. The Boolean operator “and” will be used to link the three concepts together. A draft search strategy for Ovid MEDLINE is provided as Additional file 2.

### Study records

#### Data management

This systematic review will use Covidence, an online systematic review tool. The search results from each electronic database search will be first exported into a Research Information Systems file, and then uploaded to Covidence. The Covidence system will automatically remove any identified duplicate records. Any duplicate records not identified automatically by Covidence will be removed by the reviewers. Covidence will be used to screen abstracts and to review full-text articles. If multiple different reports detailing the same study have been published, then the most recently published report will be used. If the same study has been published as both an abstract and full paper, then the full paper will be used. We also plan to use the Covidence system for data extraction and for recording of extracted data.

#### Selection process

Screening and assessment of full-text studies will be carried out using Covidence. The titles and abstracts identified through the electronic search will be screened independently by two reviewers (DNJ and either RCT or HGC). This will select potentially eligible records for full-text review. These full-text records will then be independently screened by two reviewers (DNJ and either RCT or HGC) against the pre-specified inclusion criteria. Disagreements at either stage will be resolved by discussion, or if they cannot be resolved by discussion, then by an independent review by a third reviewer (HGC or RCT). Decisions made at the abstract screening and full-text screening stages will be recorded on Covidence. If, after full-text screening, the decision is made not to include a study, the reason for this will be recorded. A flow diagram will be produced to show the flow of studies in the systematic review, from the initial search through to inclusion in the final review [39].

#### Data collection process

Data from full-text records will be extracted using a pre-defined form and will be recorded using Covidence. The data extraction form will initially be piloted on a small number of studies, to ensure that it is capturing all of the necessary data. Data will be extracted by one reviewer (DNJ), and checked by a second reviewer (either RCT or HGC). Disagreements will be resolved by discussion, or if they cannot be resolved by discussion, then by an independent review by a third reviewer (either HGC or RCT). If all of the required data is not obtainable from the full-text records, the original study authors will be contacted where necessary, using a maximum of two email attempts.

### Data items

The following information will be extracted from each of the individual studies: author names and date of publication; study setting; study population, participant demographics and baseline characteristics; biomarkers assessed as well as the timing and method of assessment and sample type; study design and methodology; recruitment and study completion rates; outcomes of interest and timing of measurement.

### Outcomes and prioritisation

The primary outcome is the risk of progression of BO which is non-dysplastic, has low-grade dysplasia, or is indefinite for dysplasia to high-grade dysplasia or OAC. Studies must assess the epigenetic biomarker(s) in patients with BO prior to subsequent progression to high-grade dysplasia or OAC, and should exclude patients who had high-grade dysplasia or OAC at baseline. The secondary outcome is the level of particular epigenetic biomarkers in patients with BO who progressed to high-grade dysplasia or OAC compared with those who did not progress.

The exact outcome measures and statistics which can be determined will depend on the data which is presented in the included studies. For the primary outcome, possible measures of effect are sensitivity, specificity, positive predictive value, negative predictive value, area under the receiver operating characteristic curve, risk ratio, and odds ratio associated with using a particular biomarker or group of biomarkers to predict progression. For the secondary outcome, possible measures of effect are likely to include mean differences in the levels of epigenetic biomarkers between patients who progressed and patients who did not progress, potentially assessed by Student’s *t*-tests or Mann–Whitney *U* tests.

### Risk of bias in individual studies

The Quality in Prognosis Studies (QUIPS) tool will be used to assess the risk of bias in included studies [40]. This tool consists of six domains: study participation, study attrition, prognostic factor measurement, outcome measurement, study confounding, and statistical analysis and reporting [40]. This will be used as a discussion point in the narrative synthesis, and may also be used to inform sensitivity analyses for any meta-analyses which are performed. The risk of bias for each study will be assessed independently by two reviewers (DNJ and either RCT or HGC). Disagreements will be resolved by discussion, or by independent assessment by a third reviewer (HGC or RCT) if necessary.

### Data synthesis

It is anticipated that the studies eligible for inclusion in the systematic review may be clinically heterogeneous in terms of the biomarkers assessed and effect measures provided. If at least three eligible studies report risk of progression associated with a particular epigenetic biomarker or group of biomarkers, then random effects meta-analyses will be used to calculate a summary risk of progression estimate associated with that biomarker or group of biomarkers, provided that the same effect measures are provided or can be calculated. Studies included within the same meta-analysis will use the same effect measure, for example odds ratio, relative risk, or hazard ratio. Meta-analysis will only be considered where studies are judged sufficiently similar in terms of outcome definition, study population and other relevant methodological characteristics. Studies judged to be substantially different clinically or methodologically may instead be synthesised narratively. Forest plots will be produced to illustrate any meta-analyses which are performed. We plan to use R package meta to perform any meta-analyses. The chi-squared statistic will be used to test for heterogeneity, and the *I*^2^ statistic will be used to quantify any heterogeneity. A *P* value < 0.10 will be considered significant for the chi-squared statistic [41]. The *I*^2^ statistic will be interpreted according to the guide in the Cochrane Handbook for Systematic Reviews of Interventions [41]. If there is a sufficient number of studies within any meta-analysis performed, then heterogeneity may be explored using subgroup analyses or meta-regression. Possible sources of heterogeneity will also be discussed narratively. Sensitivity analyses may also be performed, for example by performing any meta-analyses with and without any outlying studies, or with and without studies judged to be at a high risk of bias.

If a meta-analysis is not possible, then a narrative synthesis of the data will be carried out, following the reporting guideline for synthesis without meta-analysis (SWiM) [42]. We plan to organise the synthesis by the epigenetic biomarkers examined. Biomarkers may also be grouped by class for synthesis. Synthesis methods such as summarising effect estimates, combining *P* values, and vote counting based on direction of effect may be used, depending on the data available. Data will be presented in tables, and graphically if possible. If the necessary data are available, we also plan to perform subgroup analyses of patients with non-dysplastic BO, and patients with BO which has low-grade dysplasia or is indefinite for dysplasia. Should sufficient data be available, we may also perform subgroup analyses based on study design, follow-up duration, and biomarker assay method. If further information is required regarding the statistics presented in any of the publications, then the study authors will be contacted using a maximum of two email attempts.

### Meta-biases

We will assess the risk of bias due to missing results in the syntheses in our systematic review using the framework described in the Cochrane Handbook for Systematic Reviews of Interventions [43]. Studies will be assessed for selective non-reporting of results, for example by comparing studies’ methods sections with their results sections, and by comparing studies’ reports with any available protocols [44]. We will consider whether there are any qualitative signals of potentially missing results [44]. If there are at least 10 studies in any meta-analyses performed, then funnel plots will be produced to investigate for small study effects [45]. The Egger test will be used to assess for funnel plot asymmetry [46].

### Confidence in cumulative evidence

The Grading of Recommendations Assessment, Development and Evaluation (GRADE) methodology will be used to assess the certainty of the evidence for each outcome [47]. The GRADE approach categorises the quality of evidence into one of four levels: high, moderate, low, and very low [48].

## Discussion

This systematic review is addressing an important and clinically relevant question. With the rising incidence of BO and OAC in the Western world [8], the poor prognosis associated with OAC [49], and the late stage of diagnosis of most cases of OAC [11], there is an urgent need to identify biomarkers which could risk-stratify patients with BO. As discussed above, the current surveillance method for patients with BO, consisting of regular endoscopic examinations with biopsies being taken [1, 19, 20], has several potential limitations. In theory, clinical implementation of such biomarkers could allow patients deemed to be at high risk of progression to undergo more intensive surveillance, to maximise the chance that any OAC that develops is detected at an early stage. Additionally, patients deemed to be at low risk of progression could have surveillance intervals extended or potentially discontinued. In addition to potential clinical applications, identification of relevant biomarkers may also improve our understanding of the molecular biology underlying the transition from BO to OAC. This systematic review also has the potential to identify important areas for research in the future.

Scoping searches detected one previous systematic review published in 2018 which had investigated epigenetic biomarkers in the progression from non-dysplastic BO to OAC [50]. We believe our systematic review to be necessary and justified. Whilst on a similar topic, the exact question that our systematic review is examining will be broader in terms of the population being studied. The previous systematic review was looking at progression specifically of non-dysplastic BO to high-grade dysplasia or OAC. Our systematic review will examine the progression of BO that is non-dysplastic, has low-grade dysplasia, or is indefinite for dysplasia to high-grade dysplasia or OAC. Additionally, epigenetics is a fast-moving field, with a large volume of research regularly being published. The use of biomarkers in the screening for and surveillance of BO is also highly topical and an area of active research. Given this previous systematic review was carried out 8 years ago, there is a definite need to review the current state of evidence on this topic. For example, our draft search strategy for Ovid MEDLINE (provided in Additional file 1) returned 148 potentially relevant results subsequent to the publication of the 2018 systematic review. Furthermore, we will adopt our own approach to the search strategy, study screening, data extraction, risk of bias assessment, and data synthesis, which may yield different search results, as well as novel interpretations and conclusions, thus adding value to the published literature in this field. Our review will therefore be an entirely new systematic review, rather than simply an update of the previous similar one.

The strengths of this systematic review include an organised protocol developed in accordance with the PRISMA-P statement, a comprehensive search strategy aiming to identify all relevant studies, and a structured and thorough approach to assessments of bias and data synthesis. In terms of limitations, we anticipate that the studies eligible for inclusion will be clinically heterogeneous, for example in terms of epigenetic biomarkers assessed and outcomes studied. This may preclude a meta-analysis from being performed. Additionally, it is possible that some sources of grey literature may not be identified by our search strategy. We intend to disseminate our findings by submitting the completed systematic review for publication in a peer-reviewed journal.

## Supplementary Information

Additional file 1: PRISMA-P 2015 Checklist. A populated PRISMA-P checklist, indicating whether each piece of information is reported or not, and which line number(s) it is reported in.

Additional file 2: Draft search strategy for Ovid MEDLINE. A draft search strategy for the database Ovid MEDLINE.

## Data Availability

Not applicable.

## Abbreviations

BO: Barrett’s oesophagus
GRADE: Grading of Recommendations Assessment, Development and Evaluation
OAC: Oesophageal adenocarcinoma
PRISMA: Preferred Reporting Items for Systematic Reviews and Meta-Analyses
PRISMA-P: Preferred Reporting Items for Systematic Review and Meta-Analysis Protocols
SWiM: Synthesis without meta-analysis
