# Supplementary material for: Epigenetic biomarkers and the progression of Barrett’s oesophagus to oesophageal adenocarcinoma: a systematic review protocol
Source: Syst Rev. 2026 Jul 8;15:258. doi: 10.1186/s13643-026-03256-0 (PMC13629058; doi:10.1186/s13643-026-03256-0)
Supplement: Supplementary file 2 [file 13643_2026_3256_MOESM2_ESM.docx]

**Draft search strategy for Ovid MEDLINE:**

1. Barrett Esophagus/ or barrett esophagus.mp. or barrett oesophagus.mp. or barrett's oesophagus.mp. or barrett's esophagus.mp.

2. Biomarkers/ or biomarker*.mp. or biological marker*.mp. or Biomarkers, Tumor/

3. Epigenomics/ or epigenomic*.mp. or epigenetic*.mp.

4. methylation/ or dna methylation/ or methylat*.mp. or DNA methylat*.mp.

5. Histones/ or histone*.mp. or histone modification*.mp. or histone methylat*.mp. or histone acetylat*.mp.

6. Chromatin/ or chromatin*.mp. or "Chromatin Assembly and Disassembly"/ or chromatin remodelling.mp.

7. epigenesis, genetic/ or chromosomal position effects/ or dosage compensation, genetic/ or x chromosome inactivation/ or epigenetic memory/ or epigenetic repression/ or gene silencing/ or crispr-cas systems/ or rna interference/ or genomic imprinting/

8. rna, untranslated/ or rna, catalytic/ or rna, long noncoding/ or rna, small untranslated/ or micrornas/ or rna, guide, crispr-cas systems/ or rna, guide, kinetoplastida/ or rna, small cytoplasmic/ or rna, small interfering/ or piwi-interacting rna/ or rna, small nuclear/ or rna, small nucleolar/ or rna, spliced leader/ or regulatory sequences, ribonucleic acid/ or internal ribosome entry sites/ or riboswitch/ or untranslated RNA*.mp. or long non-coding RNA*.mp. or lncRNA*.mp. or non-coding RNA*.mp. or ncRNA*.mp. or small interfering RNA*.mp. or siRNA*.mp. or microRNA*.mp. or miRNA*.mp. or small non-coding RNA*.mp. or sncRNA*.mp. or piwi-interacting RNA*.mp. or piRNA*.mp.

9. Esophageal Neoplasms/ or Esophageal neoplas*.mp. or oesophageal neoplas*.mp. or oesophageal cancer*.mp. or esophageal cancer*.mp. or oesophageal carcinoma*.mp. or esophageal carcinoma*.mp. or oesophageal adenocarcinoma*.mp. or esophageal adenocarcinoma*.mp. or oesophageal tumo*.mp. or esophageal tumo*.mp. or cancer* of the oesophagus.mp. or cancer* of the esophagus.mp. or cancer* of oesophagus.mp. or cancer* of esophagus.mp. or carcinoma* of the oesophagus.mp. or carcinoma* of the esophagus.mp. or carcinoma* of oesophagus.mp. or carcinoma* of esophagus.mp. or adenocarcinoma* of the oesophagus.mp. or adenocarcinoma* of the esophagus.mp. or adenocarcinoma* of oesophagus.mp. or adenocarcinoma* of esophagus.mp. or tumo* of the oesophagus.mp. or tumo* of the esophagus.mp. or tumo* of oesophagus.mp. or tumo* of esophagus.mp.

10. carcinogenesis/ or cell transformation, neoplastic/ or cocarcinogenesis/

11. disease progression/

12. (high grade dyplas* or hgd).mp.

13. (Barrett carcinoma* or Barrett's carcinoma* or Barrett cancer* or Barrett's cancer* or Barrett adenocarcinoma* or Barrett's adenocarcinoma*).mp.

14. 2 or 3 or 4 or 5 or 6 or 7 or 8

15. 9 or 10 or 11 or 12 or 13

16. 1 and 14 and 15

17. limit 16 to humans

18. limit 17 to "review"

19. 17 not 18
